# Adapting a codesign process with young people to prioritize outcomes for a systematic review of interventions to prevent self‐harm and suicide

**DOI:** 10.1111/hex.13479

**Published:** 2022-05-06

**Authors:** Sarah Knowles, Vartika Sharma, Sarah Fortune, Ruth Wadman, Rachel Churchill, Sarah Hetrick

**Affiliations:** ^1^ Centre for Reviews and Dissemination University of York York UK; ^2^ Department of Psychological Medicine, School of Medicine The University of Auckland Auckland New Zealand; ^3^ Children and Young People Satellite, Cochrane Common Mental Disorders The University of Auckland Auckland New Zealand; ^4^ Department of Social and Community Health, School of Population Health University of Auckland Auckland New Zealand; ^5^ Department of Health Sciences University of York York UK

**Keywords:** codesign, evidence synthesis, outcomes, self‐harm, suicidal behaviour, youth mental health

## Abstract

**Background:**

Research and clinical outcomes that matter to people with lived experience can significantly differ from those outcomes studied by researchers. To inform a future Cochrane review of suicide and self‐harm prevention interventions, we aimed to work with young people with relevant lived experience to agree on priority outcomes.

**Design:**

Four participatory codesign workshops were completed across two sites (New Zealand, United Kingdom) with 28 young people in total. We iteratively adapted the methods over the course of the study.

**Results:**

‘Improved coping’ and ‘safer/more accepting environment to disclose’ were the final top‐rated outcomes. ‘Reduction of self‐harm’ was considered a low priority as it could be misleading, stigmatizing and was considered a secondary consequence of other improvements. In contrast to typical research outcomes, young people emphasized the diversity of experience, the dynamic nature of improvement and holistic and asset‐based framing. Methodologically, dialogue using design materials (personas) to thematically explore outcomes was effective in overcoming the initial challenge of disparate quantitative ratings.

**Discussion:**

The results will directly inform the development of a Cochrane review, enabling identification of whether and how outcomes of most importance to young people are measured in trials. Rather than producing discrete measurable outcomes that could be easily added to the systematic review, the young people challenged the academic conceptualization of outcomes, with implications for future evidence synthesis and intervention research, and for future codesign.

**Patient or Public Contribution:**

Young people with lived experience were codesigners of the outcomes, and their feedback informed iterative changes to the study methods.

## 1 INTRODUCTION

Self‐harm (self‐inflicted injury or self‐poisoning irrespective of the method or degree of intent to die) is relatively common affecting up to 25% of young people^1^, ^2^, ^3^ and rates have been increasing over recent decades across a number of countries.^3^, ^4^, ^5^ Apart from reflecting significant underlying distress, it is associated with a range of adverse outcomes, including mental health morbidity, poorer education and employment outcomes and overall decreased quality of life, as well as being costly to treat, and ultimately is associated with a higher risk of mortality including suicide.^6^, ^7^, ^8^, ^9^ Providing intervention and support to those who engage in self‐harm is, therefore, an important component of suicide prevention and a priority area for improving the mental health of young people.

The provision of support must take account of those who may never engage with clinical services. There is a widespread public and political belief that educational settings are a logical and appropriate place to provide prevention and treatment and a growing expectation that wellbeing support will be provided in these settings.^10^, ^11^ Therefore, a comprehensive high‐quality systematic review of suicide and self‐harm prevention interventions in all educational settings is being undertaken to enable evidence‐informed decision‐making for investment in prevention efforts in educational settings.

The involvement of end users in the production of reviews is recommended to increase the likely relevance and usefulness of the final review output.^12^ This aligns with agendas for health research nationally and internationally that advocate for the increased involvement of patients. Involvement in systematic reviews has been recommended as especially important given their high status within evidence hierarchies.^13^

Such involvement, to date, has been largely limited to consultative review of researcher‐authored protocols, with ‘upstream’ involvement in topic prioritization and outcome identification less common.^14^ Activities to produce consensus on priority outcomes has become more common for use in randomized controlled trials (RCTs)^15^ and in research on patient‐reported outcome measures,^16^ where outcomes of most importance to patients and carers are found to differ from those suggested as most important by clinicians and researchers.

In regard to self‐harm, there may be a considerable divergence between professional and patient priority outcomes.^17^ This has been demonstrated in studies examining what young people believe might be effective interventions, for example, two studies of young people who self‐harm using content analysis found that support from family and friends could encourage them to stop self‐harm, and it appeared more relevant to them than care or therapy.^18^, ^19^ Evidence suggests the importance of involving young people directly both in priority setting activities^20^, ^21^ and in systematic reviews. Involving young people directly in the development of a review of school interventions and health was found to have numerous benefits, including supporting researcher confidence in the choice of focus and identifying potential evidence gaps.^22^

Despite an increase in patient involvement activity occurring in systematic reviews, more explicit descriptions of the nature and impacts of such involvement are recommended.^23^ There is also a need for examination of the stakeholder involvement methodologies used in reviews.^24^ For example, approaches to prioritization often seek consensus through quantitative group ranking or voting methods. These favour broad statements which may lose the distinctiveness or subtlety of issues.^25^ It has been found with young people, for example, that submissions to priority‐setting, which are deemed ‘out of scope,’ may, in fact, contain important themes regarding what matters to young people themselves.^26^ Alternatives include more dialogue‐based approaches^27^ or participatory codesign methods.^28^ Facilitated discussions with interactive activities may be particularly suitable for working with young people.^29^

To inform the systematic review of suicide and self‐harm prevention interventions in education settings, we aimed to work with young people to collaboratively prioritize review outcomes. We adopted an iterative approach to eliciting and exploring outcomes with young people, to enable adaptation of our methodology in response to challenges encountered. This paper reports both the final outcomes produced and our learning regarding methods to support meaningful codesign. We address the following research questions:

### 1.1 Research questions

1. What methods are helpful in supporting young people to generate and prioritize outcomes?
2. What outcomes for a review of interventions in educational settings for self‐harm are important to young people with lived experience of self‐harm?
3. How do the outcomes prioritized compare to typical review outcomes, and can those outcomes be incorporated into a systematic review?

## 2 MATERIALS AND METHODS

### 2.1 Sample

#### 2.1.1 Who we involved

Across both sites we invited young people with relevant lived experience of both or either individual experience of self‐harm or indirect experience of self‐harm or suicide as a friend or family member. This was made clear in study materials and we did not seek or expect individual disclosure.

#### 2.1.2 How we involved them

We approached young people we had worked with before or sought new contributors through organizations who worked directly with young people. This was considered necessary given the topic, to ensure young people were linked with networks or support and had familiar contacts to engage with if they became distressed. Young people in both sites received reimbursement as a voucher.

In New Zealand, recruitment was via the professional networks of the researchers (S. H. and S. F.), as well as via Youth Horizons Trust, a national charitable organization in New Zealand providing support to young people experiencing different problems. The Trust invited the youth advisors within their network to participate in the workshops. A total of 13 contributors (five males and eight females) were included aged between 16 and 23 years and from diverse ethnic groups; six belonged to the indigenous population of New Zealand (Māori), four were New Zealand Europeans and four were of Asian ethnicity.

In the United Kingdom, we worked with Leaders Unlocked, an organization that supports consultation and coproduction exercises between young people and professional organizations. Fifteen young people completed a workshop pretask, eight participated in Workshop 1 and two participated in Workshop 2 (10 in total). All were aged between 18 and 23 years. Eleven were female, three were male, one nonbinary. Ethnicity was not recorded.

### 2.2 Design

The over‐arching approach was participatory codesign with an iterative methodology allowing us to learn and adapt during the process. Participatory codesign is a collaborative approach that encourages the sharing and blending of different kinds of knowledge (e.g., recognizing lived experience of mental health problems as a valuable form of knowledge and expertize).^30^ We use the term codesign as it has been used in applied health research, to describe ‘collective creativity…working together in the design development process’.^31^ In this setting, the object being designed was the review itself, and we were collectively working with young people themselves to design what parts of the review should look like or include. Codesign is a practice where people collaborate or connect their different knowledge to carry out a design task.^32^ Both codesign and coproduction are seen as examples of ‘Doing with’ (as opposed to ‘Doing to’ and ‘Doing for’), but while codesign can involve sharing of decisions (the design choices that are made), coproduction goes further to involve people in delivery.^33^ In this case, that would be an involvement in the review process itself, and here we describe involvement in decisions about the review.

Four workshops were completed overall, the first two in New Zealand (December 2019) and the second two in the United Kingdom (June 2020), as the main two locations of the Cochrane Common Mental Disorders Children and Young People's Satellite activities. The staged approach to timing allowed for reflection on outcomes and adaptations of the methods between them.

Table 1 summarizes the methods used, including why adaptations were made in response to challenges in Workshops 1 and 2.

**Table 1:** Changes made over the course of the study

	Workshops 1 and 2	Challenges identified	Adaptations in response, used in Workshops 3 and 4
Elicitation of outcomes	Presentation and discussion of systematic review research	Contributors tended to focus on interventions rather than outcomes	Persona activity (describing an example of a young person experiencing self‐harm) sent to young people before the workshop to generate outcomes
Materials, definition and method for generation	Outcomes defined as: ‘A way to understand if an activity has been effective in achieving a desired result’	The discussion was not very activity‐based, making it too didactic for young people to engage and focus on the discussion	Outcomes defined as: ‘We would like you to think about [the personas] perspective as if we are seeing them again after a service of some kind has happened at their school or college and they are feeling and doing better than they were’
	Contributors proposed a range of outcomes and discussed researcher predefined outcomes (commonly reported in such trials)	Discussion led to a large number of individual outcomes being generated	Based on responses, the researcher generated six thematic outcome categories grouping reported individual outcomes
Prioritization of outcomes	Each participant was required to individually choose three outcomes that must be included and three that must be excluded for the final list of outcomes (‘must exclude’ was included to try to narrow down the large list of individual items)	Ranking led to a significant spread of votes across outcomes for the three ‘must includes’ and three ‘must excludes’. Focus on individually prioritized outcomes meant there was overlap across these lists; that is, some outcomes were included in both lists	Trello board was used in the online workshop to collectively discuss and refine the researcher‐generated themes based on the outcomes generated, including merging themes or creating new themes were necessary
			Range voting to prioritize the order of collectively agreed outcomes

In the following section we describe the materials used, data collected and analysis performed in each workshop.

### 2.3 Data collection and analysis

#### 2.3.1 NZ workshops

##### 2.3.1.1 Data collection

Workshops 1 and 2 were facilitated by V. S. and S. H. and involved (1) a brief presentation to orient young people to what research and systematic reviews, including opportunity for questions; (2) a discussion about what activities/interventions were useful in preventing self‐harm or suicide to orient them to the types of interventions used in this area. A list of potential outcomes was generated from this discussion (in the second workshop, outcomes generated from the first workshop were also presented and discussed). Added to this was a list of outcomes that are typically reported in RCTs in this area but not mentioned by the contributors, which they were invited to keep or discard.

Prioritization of a final list of outcomes was undertaken by asking each participant to individually identify three outcomes for each of two categories—‘must include’ outcomes and ‘must exclude’ outcomes. Contributors were told that they need not discuss their choice and were asked to write their three outcomes anonymously on post‐it notes. The post‐it notes were collected and then displayed on the whiteboard, which could be then seen by other group members and facilitators, and allowed for final discussion.

##### 2.3.1.2 Analysis

Key ideas from the discussion were recorded on flipcharts and whiteboards so that contributors could see them, allowing ongoing sense‐making between the contributors and research facilitators. It also allowed iteration as contributors were able to reflect on their initial ideas and refine them as these ideas were discussed more. Data collected during these workshops were thematically analysed.

#### 2.3.2 UK workshops

Although originally planned as face‐to‐face workshops in March 2020, social distancing requirements due to COVID‐19 led to us adopting the process to work remotely in July 2020.

##### 2.3.2.1 Data collection

2.3.2.2

*Preworkshop*: In response to challenges observed in the NZ workshops, the UK workshops used a persona activity to support the generation of outcomes before the workshops.

The pretask presented two personas. Personas are brief character narratives that provide an exemplar service user who can be used as a prompt for generating ideas.^34^ They were designed to reflect different attributes of someone who may self‐harm (one involved a school‐going person and the other someone at college, one described suicidal thinking and the other self‐harm behaviour, and one included reference to the disclosure and help‐seeking and the other did not (Appendix S2). The personas were based on published qualitative research on young people's experiences of self‐harm^35^, ^36^, ^37^ and cross‐checked with three coauthors with experience of working with such young people clinically and academically (S. H., S. F., R. W.).

Contributors were asked to imagine the character in the persona after they had received support and say what would be different and how they would be doing better. The persona task was completed via email or via discussion with a Leaders Unlocked facilitator who provided verbatim notes to the research team.

All individual contributions (from 10 contributors in Workshop 1 and 5 submitted for Workshop 2) were reviewed by a single author (S. K.) who conducted an inductive and descriptive analysis to identify repeated phrases or descriptions, and group these into preliminary thematic categories, which would be shared with the group as a whole in the Workshop session. The thematic categories were also reviewed by the NZ team (V. S., S. H., S. F.) to corroborate whether they also sufficiently captured the outcomes proposed in the NZ workshops or whether additional categories should be proposed to incorporate those findings.

*Workshop 3* (first UK workshop): Workshop 3 was led by S. K. and facilitated by leaders unlocked with eight of the pretask contributors (two were unable to attend due to sickness/clashes with shift working). Each of the thematic categories identified from the pretask was presented for discussion, to explore whether the young people agreed with the grouping of outcomes into the categories, whether there was overlap or if new groups were required and agree whether any important outcomes were being missed by the categories. These discussions led to a reclassification into six outcome themes, representing a collective agreement between the researcher and young people and among young people themselves, which were then prioritized in order through range voting.

The workshop was held online using Zoom. We used Trello to visually present each of the thematic categories on digital cards (which appear as columns) and then provide examples from the pretask responses as bullet points on the cards. The cards could be moved and the bullet points updated during the discussion and ranking exercise, to demonstrate visually to contributors how their feedback was influencing the discussion and provide a visual record of the final ranking.

*Workshop 4*: Leaders unlocked organized a second workshop where contributors were asked to specifically reflect on the prioritization output from Workshop 3.

Two contributors attended the Zoom workshop (three contributors were unable to attend due to sickness/caring responsibilities); they had been members of the MH2K project on mental health in young people and were able to reflect both from their individual experience and drawing on their expertize and experience gained through the MH2K project (http://leaders-unlocked.org/publication/mh2k-2018/).

Firstly, the thematic categories from the pretask were reviewed (the outcomes from the additional five persona tasks were all covered under the previous themes and no new themes were created). Secondly, the order of ranking and perspectives about why that priority order had emerged were discussed along with how this contrasted with the outcomes typically reported in research. Finally, how these prioritized outcomes could be measured within studies was discussed.

##### 2.3.2.3 Analysis

The text responses to the persona task were inductively and descriptively analysed by a single researcher before the workshops to produce thematic categories. These were then revisited within the third workshop to engage in a collective ‘analytic conversation’^38^ with contributors to produce outcome themes. These collaborative outputs were the basis for Workshop 4. Data were generated and recorded via written feedback in response to the persona task, verbal comments captured within Trello, and field notes recorded by the workshop facilitator (S. K.). Analysis was therefore concurrent and iterative, consistent with the NZ approach.

##### 2.3.2.4 Overall analysis

The research team held four consensus meetings on Zoom to enable both UK and NZ team members to reflect on the findings and agree on key learning points, including review of documentary evidence (workshop protocols, email and meeting discussions) to consider iterative changes made.

### 2.4 Ethics

In NZ, ethics approval was obtained from the University of Auckland Human Participant Ethics Committee (022884 and 022721). In the United Kingdom, involvement activity does not require research ethics approval, and in the experience of the authors it may in fact be discouraged by contributors due to a tendency for researchers to then view contributors as ‘participants’ rather than partners, which can restrict the scope for flexibility and adaptation in response to feedback. However, due to us sharing the results of the research with an international team, and that the NZ team did require ethical approval, we obtained approval from the University of York Health Sciences Research Governance Committee (HSRGC/2019/364).

In both the NZ and UK settings, we aimed to ensure that (1) the young people had access to appropriate support if the workshop content caused any distress, and (2) that the workshops were appropriately facilitated and conducted in a sensitive and respectful way. In both settings, we worked with youth networks to provide support; the researchers who facilitated the workshops were experienced in facilitating codesign in mental health research, and we established ‘ground rules’ regarding workshop interaction. This included making it clear that young people need not disclose personal experiences unless they wished to do so, respecting confidentiality, being free to take a ‘time out’ at any time if needed but informing the facilitators by chat or text if so, and being respectful of each other's contributions. We made clear that these ‘ground rules’ applied to facilitators and the researcher as well as the young people.

In the first NZ workshop, young people were offered support by experienced youth workers from Youth Horizon Trust and by one of the clinical psychologists in the team (S. H.) for the second workshop. Debriefing before ending the workshop allowed participants to reflect on the discussions throughout the day and allow time to acknowledge how they were feeling. This was a way to identify any signs of distress and if any intervention was required. The meetings closed with some informal interaction among participants, youth workers and researchers (over coffee), which provided an additional opportunity to informally interact with participants to get their feedback about the methodology and also to ensure participants were not distressed. In the United Kingdom, support after the workshop involved follow‐up telephone calls by the Leaders Unlocked facilitators. In the NZ workshops, attendees were provided with the contact details of helpline numbers that provided wellbeing support.

## 3 RESULTS

We present the results for each Research Question in turn.

*RQ1: What methods are helpful in supporting young people to generate and prioritize outcomes?*

The challenges that we identified and our consequent adaptations are summarized in Table 1. The challenges were (a) discussion focused on interventions at the expense of outcomes, (b) didactic discussion of the existing research literature and (c) heterogeneity in voting for discrete outcomes. We revised our approach to introduce narrative personas to encourage (a) focus on the future state of the young person, to emphasize outcomes, (b) interactive discussion focused on experience rather on research literature and (c) thematic grouping of outcomes and voting through discussion rather than quantitative tallies of individual outcomes.

The adaptations made were successful in enabling us to codesign a final prioritized list of outcomes that were important to the young people.

*RQ2. What outcomes for a review of interventions in educational settings for self‐harm and suicide prevention are important to young people with lived experience of self‐harm?*

1. NZ workshops results

3.1

The outcomes identified during the two NZ workshops broadly focused on ways to measure utilization of existing mental health services, such as adherence and attendance, frequency of screening of mental health (indicating mandatory screening to avoid stigma) and delays in getting services. Contributors emphasized the need to measure how ‘youth friendly’ the services were—such as the provision of peer support, services in nonclinical settings and nondiscriminatory and stigmatizing approaches to care and treatment, including understanding the implications of breach of confidentiality on young people's success in their academic pathway (e.g., getting scholarships, becoming members of expert committees etc.). Although insightful, this tended to focus on the nature of the interventions offered.

In terms of outcomes, contributors discussed capacity‐building gains, such as improved knowledge/awareness about mental health, ability to identify signs of distress (both for self and peers) and how to seek support.

They were concerned with understanding how approachable and helpful those in their support system would be if they disclosed distress. Contributors highlighted a range of outcomes about feeling comfortable to disclose to peers, family and teachers, and including concepts like a sense of belonging and connection with other people (opposite of feeling isolated) and teachers and peers having a greater understanding of self‐harm. They also referred to broad outcomes like increasing the skills of gatekeepers, such as teachers, as a way to reduce stigma and discrimination.

The outcomes also reflected a perceived need for better ways to deal with the distress that was considered to underly self‐harm. Coping skills, particularly more specific skill‐based and behavioural outcomes like ‘doing activities that they enjoy’, having decent and reliable ways of distracting themselves and asking for help were also highlighted. Contributors emphasized self‐care (as an indicator of wellbeing) highlighting the significance of daily activities—exercise, having a daily routine, getting enough sleep or maintaining hygiene. Additionally, young people's beliefs about their own self—self‐esteem/self‐worth/self‐love/self‐respect were strongly identified by participants from both groups.

Typical outcomes from research, such as reduction in rates of students reporting suicidal ideation, did not come up in the list of outcomes identified by contributors. Contributors instead expressed how improvement is not a straightforward linear process but is more dynamic and includes milestones such as the reduction in the severity of self‐harm and might include relapse as part of this process. They noted that the idea of reduction being the goal might potentially have negative impacts, with relapse then experienced as a failure, in contrast to the more strengths‐based outcomes they had produced.

The consolidated list of outcomes from the two NZ workshops is presented in Appendix S1. Although the votes were distributed across a range of outcomes and the include/exclude list contained overlapping items, it was nevertheless observed by the research team that more outcomes in the must‐include list were framed as gains and strengths rather than reductions or deficits, emphasizing the need to focus on positive change.

2. UK workshops results

3.2

a. *Feedback to the persona and initial thematic categories*:

All contributors in the workshops agreed that the personas were credible and reflected the experiences of young people. Responses indicated significant empathizing with the young people discussed, with contributors discussing, for example, how lonely and isolated they felt without help, and how challenging it would be for them trying to change. Self‐harm was described as ‘invisible’, a form of ‘silent suffering’. Fear of other's reactions was also often emphasized, and how negative reactions could make it unlikely for the young people to seek help in the future: ‘stigma makes the anxiety worse’. ‘if it[reaction of others] escalates they don't have the space to talk about it’.

Inductive descriptive analysis of the responses by a researcher (S. K.) led to six thematic categories:

1. Reduction of self‐harm
2. Coping skills
3. Activities
4. Engaging with therapy
5. Support at school
6. Acceptance of family and peers

All of the above occurred multiple times across the pretasks (none was mentioned by only one person). The thematic categories were reviewed by the NZ team who agreed that these covered the range of outcomes generated in the initial two workshops (as presented under RQ1—1., regarding peer and teacher support, acceptance and stigma, positive behaviours, coping and engagement with mental health services).

b. *Workshop 3: Agreeing outcome themes and range voting*:
  The outcome categories were discussed and revised within Workshop 3, leading to a final list of six outcome themes which were produced collectively by the researcher and the young people. These are presented in Table 2, in order of priority, illustrated by example text from the pretask activity and with a description of how the contributors defined the outcome theme (and whether it contrasted with or absorbed the researcher‐generated categories).
  It was notable that the outcome themes were not different across the two different profiles reflected in the persona. The young people did not differentiate between suicidal thoughts and self‐harm when thinking about outcomes.
  Although almost all the pretask responses referred to the reduction of self‐harm in the future, the young people had often caveated this to argue that reduction may not clearly indicate ‘feeling better’ (as was also found in the NZ workshops). In the workshop, contributors explicitly said that they thought this would be the most important to ‘others’, such as professionals and researchers, but were keen to emphasize that it could be misleading, as people may replace one behaviour with something else and it underestimated that relapse or recurrence may be a normal part of the change.
c. *Workshop 4: Exploring measurement and contrasting outcomes*

**Table 2:** Prioritized thematic outcome categories and descriptions

Thematic Outcome	Final votes	Example quotations	Description
Better or more coping skills	10	*‘Jake will be releasing his frustration in healthier ways, it could be exercise or meditation’*. *‘Leanne will have a plan for what to do when she's upset, like watch a film or go for a walk*’.	Improved coping was seen as an asset that could be supported and which would have long‐term benefits for the young person. This included not only coping with stressors (such as relationship problems or school work) but also coping with the self‐harm itself, or with managing thoughts of self‐harm.
A safer environment, more acceptance and understanding, at home and at school	8	*‘They're not ashamed to talk about it’*. *‘Knowing they can speak to someone if they need to’ ‘[parents and teachers] don't get angry or think it's their fault’*. *‘Stigma has the potential to worsen mental health issues by large amounts’*.	In the workshop, ‘Acceptance of family/peers’ was elaborated to refer to an accepting environment where young people would feel safe to talk about self‐harm. The contributors had discussed the isolation of self‐harm for young people, due to their fears about how others would react to it. An environment where the experience and disclosure of self‐harm were normalized would reflect a reduction in this isolation and remove a barrier to help‐seeking.
Greater teacher and peer awareness of self‐harm and how to speak about it	5	*‘They'll have more understanding and they won't treat [Jake/Leanne] any differently’*. *‘A disclosure almost always results in immediate escalation and sometimes this can be quite alienating for young people coming forward about what they are going through’*.	The original theme of ‘School support’ was partly merged into ‘Acceptance’ as the discussion showed the perceived importance in this theme was of a supportive, accepting environment, but also separated into ‘Peers and teachers understanding of self‐harm’, as the young people emphasized the potential for negative reactions that could prevent young people from seeking help. The young people felt teachers specifically were not well equipped to deal with young people who self‐harm. This new theme (‘Peers and teachers understanding’) was kept separate from ‘Acceptance’ by the young people, to focus on the ways that negative interactions with peers and teachers could be detrimental.
Increase in activities that the young person enjoyed previously and more social activities with others	2	*‘They would be less withdrawn, and doing things they enjoy’*. *‘Even little things can be little victories’*.	‘*Activities*’ was adapted to specifically refer to ‘increase in activities previously enjoyed’ due to the young peoples' wish to focus on differences between individuals. They did not want to suggest that all young people should be judged on doing particular activities or doing a specific amount of activities, emphasizing that this should consider individual preferences. The contributors considered this separate to ‘coping’ (though overlapping) as it reflected a sign of ‘things going well’ to do more positive things, but coping referred specifically to how young people managed when things are hard.
Reduction of self‐harm thoughts and behaviours	2	*‘Reduced self‐harm over time. But not necessarily stopping entirely’*.	Contributors reported that if self‐harm was functioning as a coping mechanism itself, it would be wrong to expect people to stop completely before they had learned other ways of managing, and a reduction in self‐harm itself may not mean that the factors responsible for the self‐harm were reduced.
Sustaining engagement with therapy	0	*‘I hope that she's been able to find a therapist that she feels understands her’*. *‘Going to counselling at college and having a positive experience with it’*.	As with the other outcomes, the young people were keen to emphasize individual variation—sustaining therapy should be measured according to how long the young person wished to engage rather than by a standardized measure (such as the number of sessions) and would indicate they found ‘something that's right for them’.

The goal of Workshop 4 was to obtain further feedback from young people themselves about how the final ranked outcomes could be measured, and consider how and why they contrasted with typical review outcomes. Questions about measurement stemmed from the research team observation, after Workshop 3, that the outcomes reflected a processual understanding rather than being individual and discrete, for example observing that ‘doing enjoyable activities’ could be considered an output of enhanced coping, and that increased teacher and peer awareness could be a means for creating a safer, more accepting environment. This suggested the outcomes could be understood more as a logic model of different mechanisms and impacts.

In the workshop, the young people agreed with this to an extent, though were wary of trying to specify how improvement would work for different people. They also wanted to emphasize that all the outcomes were important.

Quotes from Workshop 4 offering discussion of the final ranking:

‘Reduction’ being a low priority:

*If their self‐harm is reduced, it might be that something else has got worse that you're not measuring on the scale*

*The reduction will be second to coping and feeling safer. It might happen because of them*

Therapy being a low priority:

*Therapy is not there 24/7 or would not always be there. Coping is something you use outside therapy and you can take it with you for longer*

*People know in reality that therapy is for a short time and it might not work for everyone. It can be beneficial but it should not be the only thing*

Coping being top‐ranked:

*Coping is something empowering. It is something they can do themselves*

Coping was also described as something that could be individualized: ‘It's them identifying what helps them to cope’. Contributors liked the description of a ‘toolbox’ of different ways of coping that individuals could use and that this allowed for different individuals to have different strategies.

Safer environment being top‐ranked:

*You spend the majority of your time in school so of course it can have a massive impact on them*

This ranking reflected the recognition that while increased coping would make a big difference, to get to those improvements the young people would need to feel supported to access help, and that a negative environment was a key barrier to help‐seeking.

*RQ3: How do the outcomes prioritized by the young people compare to typical review outcomes, and can their outcomes be incorporated into a systematic review?*

The research team (all coauthors) qualitatively identified through group discussion the following differences in how the young people conceptualized outcomes, in contrast to the way outcomes are typically defined by researchers within reviews. These are described below and illustrated with comments from the young people made during the workshops

1. *Diversity of experience*: Although the aim of the workshops was to produce a consensus on priority outcomes, researchers in both settings commented that the discussions highlighted ‘the variety of voices’. This was evident in how young people in both the NZ and UK workshops cautioned that ‘what works’ can be very different for different individuals, and equally what indicates they are feeling better can be very different. The young people recognized that ‘professionals’ and ‘organisations’ would want overall measures, but questioned ‘can the outcomes be measured as successful by them [young people]?’ (Workshop 3). This underlay the challenge of prioritizing, when contributors in both sites emphasized that all the outcomes could matter to different people. This individual focus was in contrast to the focus of reviews on population‐level and aggregate outcomes.
2. *Dynamic nature of improvement*: This underpinned the rejection of ‘reduction’ as an outcome. They also suggested it could have negative impacts for young people, who might then see relapse as a ‘failure’ rather than as a normal part of a recovery journey. ‘Relapse is common and doesn't erase progress’ (Pretask), ‘Recovery isn't linear it's a big squiggle on the page’ (Pretask).
3. *Holistic understanding of outcomes*: Both in the responses to the pretask and during the discussion, it was evident that young people had a holistic view on self‐harm that considered the individual themselves and their wider experiences, their relationships with family members, friends and teachers at school, and also the broader social environment.
4. *Asset‐based perspective*: In both the NZ and UK workshops, the researchers were struck by the way that the young people framed outcomes in terms of gains or benefits, which exposed a contrast with the academic review focus, which described negative symptoms or experiences to be alleviated. ‘It's really easy to see the negative that has to be removed, rather than the positive they have to build on’ (Workshop 4).

b. *Reflections on integrating the generated outcomes into the review*:

The initial project aim was to use the generated outcomes to select measurable outcomes that could be integrated into the systematic review protocol, enabling the review authors to prioritize discrete additional outcome measures resulting from the codesign process. The top‐ranked outcomes (better coping and a safer/more accepting environment) posed a challenge to this. The research team initially discussed translating the suggestions into more discrete outcomes that were likely to have been measured in the trials literature, but were concerned that this would undermine their validity and lose the complexity that young people had emphasized.

Text extracts from research team discussion:

*Should we use a measure/measures of coping e.g. positive and negative coping style, but is that what young people meant? Maybe they mean more specific concrete and behavioural things – and then do we have each and every one of these as an outcome – a potentially huge list of outcomes. Same issue with ‘safer environment’ – a domain with a whole heap of things that have been measured?*. (R1)

*Better coping' = coping strategies? Can we compare different scales of coping, or would this be viewed analytically as several different outcomes? Is ‘safer environment’ help seeking measures? Perceived stigma measures? Can we synthesise across those?*. (R2)

*Is that what they [young people] mean? Are we over‐ruling their lived experience terms with clinical terms?*. (R3)

The process exposed two anxieties. Firstly, the concern that if outcomes proposed were not ‘measurable’, then this would produce an empty review of limited usefulness to decision‐makers (as it would highlight gaps in evidence, but not identify evidence available to inform decision making), or alternatively would mean including a multitude of proxy measures, which again would have limited usefulness as they could not be synthesized. Secondly, a concern that if the academic review team selected narrow outcomes that we expected to occur in the literature, this would involve the researchers imposing an academic lens on the insights from contributors, with the risk of mistranslating or obscuring the original meaning.

Therefore, rather than trying to reduce the young peoples' input into individual outcomes, we considered how to integrate their challenge of recognizing broader and more diverse outcomes into the review itself. The review protocol was updated to include and capture potentially relevant outcomes under the domains of the two priority outcome themes (coping and safer environment), to continue the exploration of if and how these aspects can be measured.

## 4 DISCUSSION

Young people with lived experience of self‐harm had a notably broader and more complex understanding of outcomes that would indicate improvement, compared to the relatively narrow definitions employed in academic reviews. They also emphasized diversity in response and improvement and described outcomes in a more asset‐oriented way. ‘Better coping’ and ‘a safer environment to disclose’ were the final top‐ranked outcomes. These pose challenges to measurement in a typical review, and consequently, we have modified the planned review to identify and assess if and how these outcomes are reported in the literature.

The results are consistent with Owens et al.'s^17^ study exploring outcomes for trials of self‐harm interventions. Adult participants in their study similarly had reservations about the reduction of self‐harm as a misleading outcome and instead emphasized positive improvements. The greater focus on social experience and environment is also consistent with other studies of young people.^39^ We concur with Owens et al.^17^ that these findings challenge researchers in the field to move beyond a limited conceptualization of improvement as reduced frequency of self‐harm, to instead aim for a richer understanding of sustained improvement.

It was apparent throughout the study that the young people did not differentiate between self‐harm and suicidal behaviour, in contrast to the clinical and academic literature. This is consistent with their emphasis on understanding the emotions and experiences, which underlie such behaviours, rather than focusing on the behaviour itself (including not considering repetition of the behaviour to be a priority outcome). This is also consistent with the contributors' repeated emphasis on diversity of experience and change over time, in opposition to attempts to exclusively categorize young people according to the presence or absence of specific behaviours. We were mindful of checking for differences in outcomes across the NZ and UK samples, but observed consistency in the nature of the outcomes proposed. This may reflect that the young people were focused on the emotional experience of self‐harm, which may have common elements, as opposed to service provision or availability of interventions, which would be different across the two settings.

### 4.1 Implications for evidence synthesis

The results indicate that outcomes, as conceptualized by young people themselves, are significantly different from how outcomes are understood within research. Young people emphasized diversity, dynamic change and holistic complexity. This understanding contrasts with the reporting of static and aggregate measures within research studies. This underscores the need for involvement in reviews, to act as a counterpoint to the privileging of what can be measured over what should be assessed. The more challenging conceptualization held by young people suggests that more complex reviews may be required which for example integrate narrative or qualitative synthesis alongside quantitative meta‐analyses.

We have attempted to fully report our own process of being challenged by the young peoples' suggestions and the anxieties involved. We believe this provides a more authentic, and hopefully valuable, account of the challenges involved in negotiating between academic and lived experience understanding. Systematic reviews are technically precise pieces of research activity that can leave little flexibility, and the final process of integration of the young peoples' outcomes in the review was less direct than initially planned. We argue however that narrowing the young peoples' outcomes into measurable definitions, consistent with typical systematic review outcomes, would have undermined their contribution to the review, rather than being indicative of greater impact. Instead, we have embraced the challenge posed by those young people around what and how broader outcomes are measured by making this a key question of the review. This will enable us to interrogate what is included or missing from the current evidence base.^40^ Although the team was anxious to avoid an empty review, we recognize the crucial role that reviews play in identifying gaps and thereby setting future research agendas, as will likely be the case here.

We suggest that this is consistent with the role that the young people themselves performed during the study, of pushing us to consider what we may miss. While we originally hoped for a final consensus in which we could point to a single codesigned outcome in this review, the young people had a more disruptive agenda, wishing to challenge us in our thinking and resisting attempts to simplify their contributions. This paper therefore adds to work on public involvement, which emphasizes the value of constructive disagreement as a process of two‐way learning. This requires a degree of reflexivity within the research team, and this may be challenging for researchers involved in reviews, particularly if they lack a background in qualitative or participatory research. It was also time‐consuming, and future reviews should be mindful that time and collaboration are needed to work out how reviews should be influenced, as the suggestions may not be simple or straightforward to implement.

#### 4.1.1 Implications for methods of prioritization of research outcomes

Panel voting methods such as Delphi can be viewed as appealing as they negate the need for direct interaction, which can be challenging.^13^ We would argue however that this misses an opportunity for interactive dialogue and debate, which can provide a deeper level of exploration of differences between researcher and patient views. We provide evidence for Oliver et al.'s^41^ suggestion that direct interaction and discussion between young people and researchers are most likely to influence researcher perspectives. Managing such interactions however requires greater facilitation and requires consideration of how to create supportive environments.^42^ In this study, the use of established networks to support young people to become involved helped to achieve this. Researchers should consider the demands of different methods and whether they have the capacity and support to deliver them.

Methodologically, we found the Persona method to be an acceptable and valuable way of specifying which part of the review (outcomes) we wished to discuss. Future research could consider how it can be used with other review elements (e.g., intervention characteristics, populations to include or exclude). We found that dialogue‐based activities with thematic coanalysis produced clearer outcomes (both discrete outcomes and themes regarding attributes of those outcomes) compared to the voting methods used in the first workshops. While this process still did not result in individual outcomes that could be added to the review, we suggest it achieved a vital impact in terms of challenging our understanding, and in demonstrating there is not a single ‘youth voice’ that can be distilled into a discrete priority. The final impact of the codesign was not additive (producing an outcome to be considered alongside those chosen by researchers) but transformative, leading the review team to add a novel narrative element to the future review to address the issues of what different outcomes are reported and how.

Our study suggests that participatory codesign methods, which actively engage young people in dialogue about mental health are both feasible and valuable, and that it is possible to conduct such activities in a sensitive and supportive way. Members of the author team have previously encountered concern, particularly from clinicians, about engaging with young people directly to explore perceptions of self‐harm. It has been observed that professional gatekeeping around the involvement of young people in research can conflict with the imperative to respect young people's rights.^43^ A rights‐based approach, by contrast, emphasizes the right of young people to be heard and to have influence over issues that concern them including research.^44^ We suggest our study demonstrates this approach is both necessary and achievable.

### 4.2 Limitations

Although involvement does not presuppose representative experience, diversity of experience is an important concern, particularly ensuring that ‘easy to ignore’ groups are deliberately sought and supported to contribute. Young people with mental health problems can be already considered an ‘easy to ignore’ group, but future work should explicitly seek to collaborate with young people from diverse backgrounds. This was recognized as crucial in the NZ sample, which specifically sought to include young Maori contributors, but in the UK sample, males were underrepresented and we did not collect data on ethnicity. Although the online workshops delivered in the United Kingdom, due to COVID‐19 lockdown restrictions, were effective, it is necessary to consider in future work whether digital ways of working are inclusive or can exclude some young people from contributing. In this study, we have predominantly reflected on the experiences of the researchers in the process, and in the future, the impact on the young people themselves should be evaluated. It is particularly a gap in the process that young people were not involved throughout, to the stage of reporting in this paper, and we would endeavour to involve young people throughout in future work.

## 5 CONCLUSION

Young people offer a notably different view of what kind of outcomes are important to assess in self‐harm research. Codesign methods supported young people and researchers to work together to surface and explore these differences. The workshops led directly to changes in a planned Cochrane review, to seek to better understand if and how outcomes that matter most to young people have been assessed in the literature. This will be essential to challenging future research to better recognize what outcomes are meaningful to young people themselves.

## AUTHOR CONTRIBUTIONS

Sarah Hetrick, Vartika Sharma, Rachel Churchill and Sarah Fortune conceived the study. Sarah Knowles and Ruth Wadman contributed to study design. Data were collected by Sarah Hetrick, Vartika Sharma and Sarah Knowles. All authors contributed to the analysis. Sarah Knowles prepared the first draft of the paper. All authors reviewed the paper and approved the final version.

## Supporting information

Supplementary Information

## Data Availability

The data that support the findings of this study are available on request from the corresponding author. The data are not publicly available due to privacy or ethical restrictions.
