# Supplementary material for: Adapting a codesign process with young people to prioritize outcomes for a systematic review of interventions to prevent self‐harm and suicide
Source: Health Expect. 2022 May 6;25(4):1393–404. doi: 10.1111/hex.13479 (PMC9327872; doi:10.1111/hex.13479)
Supplement: Supplementary file 1 — Supplementary Information [file HEX-25--s001.docx]

Appendix 1:

Consolidated list of outcomes from the 2 New Zealand workshops (verbatim quotes):

**Must-include outcomes:**

| Self-care (such as eating, sleeping, doing hair), self-love, self-esteem, respecting yourself | 5 votes |
| --- | --- |
| No. of young people trained on identifying potential signs of mental distress among peers/Self-reporting of mental health status/Proportion of young people who feel comfortable to seek help | 5 votes |
| Teachers know how to recognise/be aware of signs of distress in students/Feeling connected/De-stigmatisation and inclusion of holistic student support services, having conversations with friends/family | 5 votes |
| Ability to cope with stress, participation in extra-curricular activities, /sense of control over self-improvement | 4 votes |
| Retention in services/Engagement with counsellor | 4 votes |
| Being hopeful about future | 3 votes |
| Positive Attitude/Behaviour (confident, happy, sound interaction) | 2 votes |
| Negative Attitude/Behaviour (substance use, self-harm) | 2 votes |
| Socio-demographic profile of those who seek services (to know what makes them access services) | 2 votes |
| Regular check-ins with young people | 1 vote |

**Must-exclude outcomes**

| Appointment of a nodal person as “mental/wellbeing coordinator” | 4 votes |
| --- | --- |
| Participation in sports or other activities | 4 votes |
| Spending time with parents/More comfortable speaking to parents about their issues | 4 votes |
| Doing things differently (learn new skills e.g. emotional regulation) compared to before (when they were not feeling well) | 3 votes |
| YP who report less motivation/less interest | 2 votes |
| Negative behaviours/attitudes (aggression, substance use) | 2 votes |
| Training of teachers/staff on mental health/suicide prevention | 2 votes |
| Positive attitude/behaviours | 1 vote |
| Reports of Suicidal ideation | 1 vote |
| No. of young people who report adherence to intervention | 1 vote |
| Connecting with peers | 1 vote |
| Ranking people who YP feel comfortable with in discussing about MH services | 1 vote |
| (Ease of) Availability of information about MH services | 1 vote |
| (Ease of) Availability of intervention at site | 1 vote |
| Number of people using services | 1 vote |
| Achieving better grades at school | 1 vote |

Appendix 2

**Persona 1**

Jake is 15 years old and goes to school in Manchester. He’s studying for his GCSEs, and thinks he will do ok in some subjects but is worried about others. He plays football on a Saturday with his brother and some other boys from his school, but otherwise tends to keep to himself most of the time and prefers being in his room on his own playing Xbox or watching shows online.

Jake has been cutting himself when he gets angry about things, for over a year now. Jake hasn’t told his parents or teachers about his self-harm. He’s worried about what they will think. He knows some other people at school who have self-harmed, and that other students say they just did it for attention and it was stupid. He feels like people won’t understand or will think that he’s weird.

He first started to self-harm when he was being bullied. The kids who were making fun of him are in a different class now, but he still self-harms sometimes. Recently he has tended to self-harm after he’s had an argument with his parents, and everything feels too much.

Afterwards he sometimes feels stupid about doing it, and feels ashamed. He has tried to think of ways to stop doing it. Sometimes it helps if he does something different, like drawing or going for a walk. But other times he doesn’t know how else he would cope.

Jake feels like he doesn’t have anyone to talk to when he gets upset and tends to keep everything to himself. He especially thinks it would too uncomfortable to talk to anyone about the self-harm.

**Persona 2**

Leanne is 17 years old and goes to college in Nottingham. She wants to be a teaching assistant, and maybe a teacher one day if she can stick with the training. She’s missed quite a lot of classes so far this year because she’s hasn’t wanted to see or speak to anyone, and just stays in bed most of the day.

Leanne was diagnosed with anxiety and depression when she was 14. She’d been struggling with sleep, and panicking at school. She felt like she couldn’t cope with everything, and that was when she would think about suicide. It made her feel better at first, like there was finally something she could control, even though afterwards she would feel bad that she had even considered it. She hasn’t spoken to anyone at school about it, not her friends or the teachers there. She thought about telling her tutor, but she was scared she would think differently about her afterwards.

Leanne had counselling in the past, but she didn’t feel like it helped. She felt like the therapist was patronizing her, and she didn’t really understand the point of the forms and tasks they went through. She doesn’t remember now if she just stopped going, or if the service discharged her. No-one has ever followed it up. Her college has a counselling service, but she’s not sure she wants to talk about it again, especially if it’s like the therapy she had before.

She told her mum and dad that she had thoughts about suicide a few years ago. Her mum got really angry about it. She hasn’t mentioned it again since then, and gets mad when her mum brings it up. She doesn’t want her Dad to know she still thinks about it sometimes, because then he’d get upset.
